# Supplementary material for: Toll-like receptor 2 expression on c-kit+ cells tracks the emergence of embryonic definitive hematopoietic progenitors
Source: Nat Commun. 2019 Nov 15;10:5176. doi: 10.1038/s41467-019-13150-0 (PMC6858454; doi:10.1038/s41467-019-13150-0)
Supplement: Supplementary file 1 — Supplementary Information [file 41467_2019_13150_MOESM1_ESM.pdf]

**Supplementary Information**

# Toll-like receptor 2 expression on c-kit$^+$ cells tracks the emergence of embryonic definitive hematopoietic progenitors

Jana Balounová, Iva Šplíchalová, Martina Dobešová, Michal Kolář, Karel Fišer, Jan Procházka, Radislav Sedláček, Andrea Jurisicova, Hoon-ki Sung, Vladimír Kořínek, Meritxell Alberich-Jorda, Isabelle Godin and Dominik Filipp

**Supplementary Figure 1**

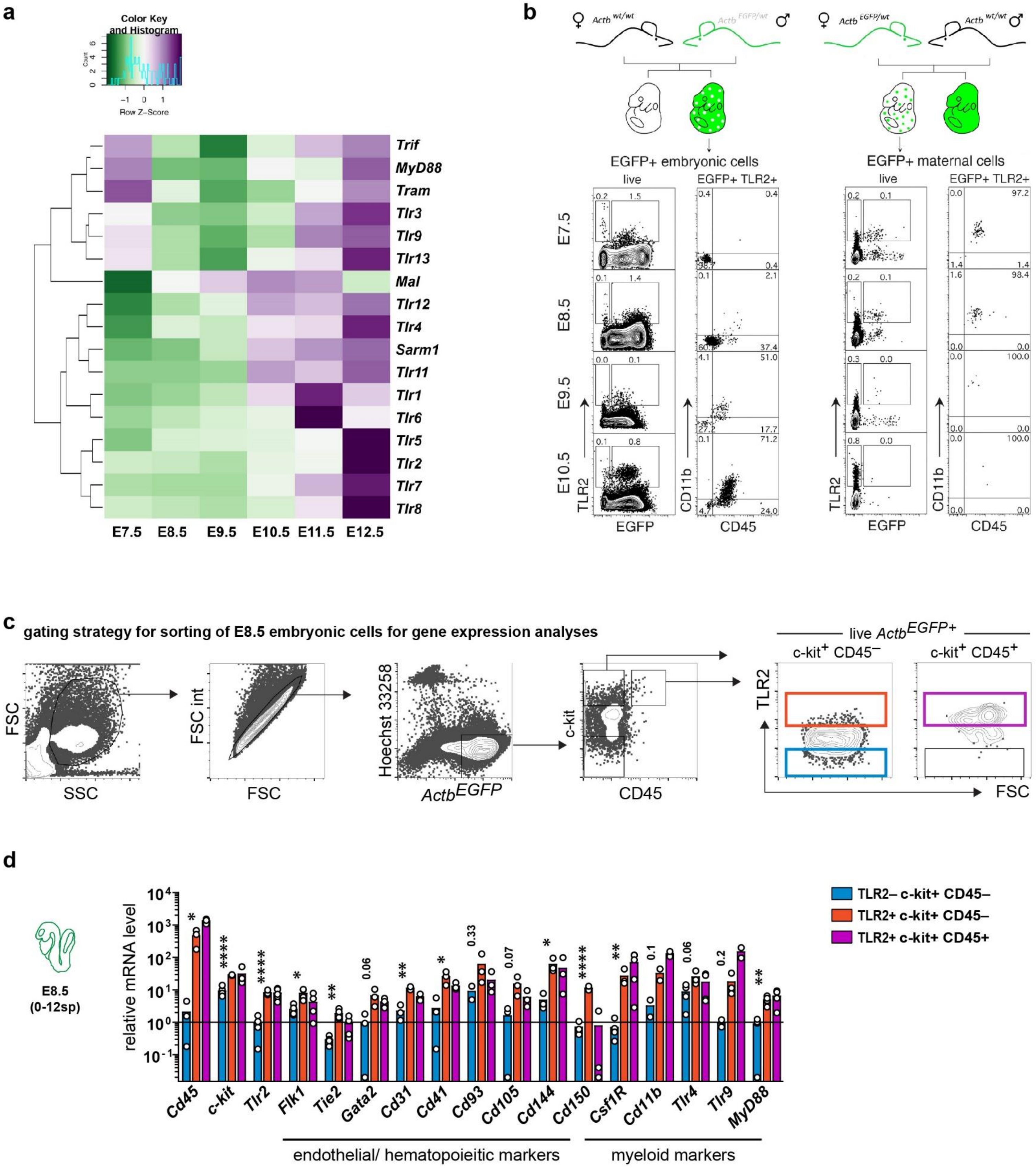

**Supplementary Figure 1**

**Embryonic TLR2$^{+}$ hematopoietic progenitors express TLRs and genes associated with EHT**

**(a)** Expression of *Tlrs* and *Tlr* adaptors during murine early embryonic development.

The heat map represents the hierarchical clustering of the normalized mean of mRNA expression levels of *Tlrs* and *Tlr* adaptor protein genes (using qPCR) at different stages of embryonic development (E7.5-E12.5). The observed signal was normalized to the mRNA levels of the reference gene *Casc3*. To generate the heat map, the gene expression was Z-score normalized after scaling the highest value to one (n=3).

**(b)** Tracking of embryonic and maternal TLR2$^{+}$ cells. Tracking of embryonic cells (two left panels): *Actb*$^{wt/wt}$ females were crossed with *Actb*$^{EGFP/wt}$ males. Only EGFP$^{+}$ (*Actb*$^{EGFP/wt}$) embryos were analyzed. Based on EGFP expression, embryonic cells were distinguished from EGFP$^{-}$ maternal cells (*Actb*$^{wt/wt}$, white dots). EGFP$^{+}$ *Actb*$^{EGFP/wt}$ embryos (E7.5-E10.5) were analyzed for TLR2, CD11b and CD45 expression. The right side panels show the expression of CD45 and CD11b on gated *Actb*$^{\mathrm{EGFP+}}$ TLR2$^{+}$ embryonic cells. Tracking of maternal cells (two right panels): *Actb*$^{EGFP/wt}$ females were crossed with *Actb*$^{wt/wt}$ males. Only EGFP$^{-}$ (*Actb*$^{wt/wt}$) embryos were analyzed by flow cytometry (FCM) at E7.5-E10.5 for the presence of transplacentally transferred *Actb*$^{\mathrm{EGFP+}}$ (*Actb*$^{EGFP/wt}$) maternal cells (green dots) by staining for TLR2, CD11b and CD45 markers. The right side panels show the expression of CD45 and CD11b on gated *Actb*$^{\mathrm{EGFP+}}$ TLR2$^{+}$ maternal cells. Representative data from three independent experiments are shown.

**(c)** Gating strategy for sorting of E8.5 *Actb*$^{\mathrm{EGFP+}}$ cells based on their c-kit, CD45 and TLR2 expression to determine their gene expression pattern.

**(d)** The relative mRNA expression (*Casc3* was used as reference gene) of endothelial, hematopoietic and myeloid genes, analyzed in sorted E8.5 embryonic cells of the indicated phenotypes, was normalized to the levels of expression in the TLR2$^{-}$ c-kit$^{-}$ CD45$^{-}$ population. Statistics are shown for comparison of gene expression among TLR2$^{-}$ c-kit$^{+}$ CD45$^{-}$ and TLR2$^{+}$ c-kit$^{+}$ CD45$^{-}$ populations (mean ± SD; n=3-5; *$p \leq 0.05$, ** $p \leq 0.01$, *** $p \leq 0.001$, **** $p \leq 0.0001$; unpaired, two tailed t-test).

Source data are provided as a Source Data file.

**Supplementary Figure 2**

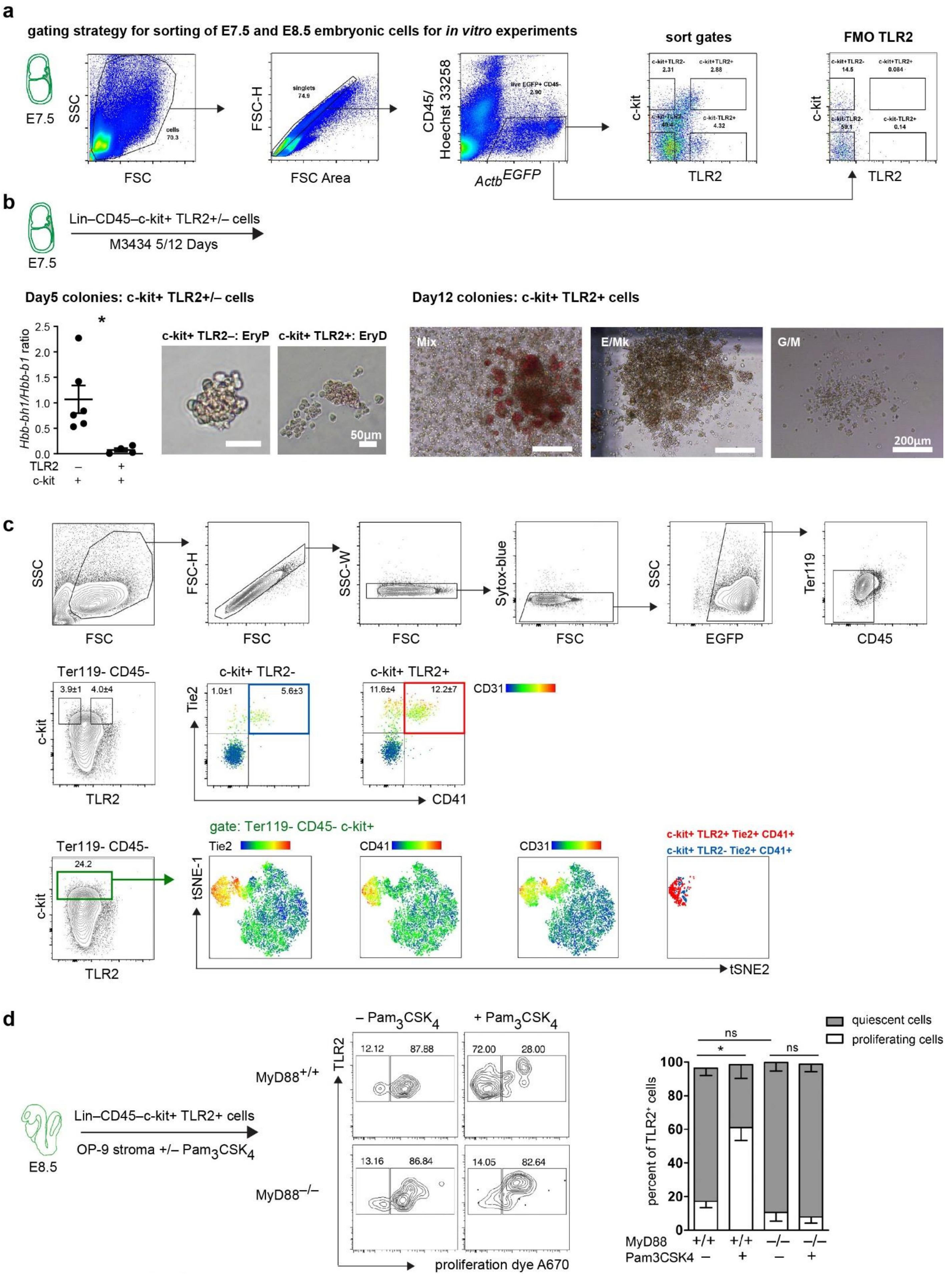

**Supplementary Figure 2**

**Embryonic TLR2$^+$ c-kit$^+$ progenitors exhibit features of EMPs and respond to TLR2 triggering**

**(a)** Gating strategy for sorting of E7.5 and E8.5 *Actb*$^{EGFP+}$ CD45$^-$ cells based on their c-kit and TLR2 expression for *in vitro* experiments in Fig. 1f, h-j and Supplementary Fig. 2b.

**(b)** E7.5 TLR2$^+$ c-kit$^+$ Lin$^-$ CD45$^-$ progenitors have erythroid, myeloid and erythro-myeloid potential. E7.5 *Actb*$^{EGFP+}$ Lin$^-$ cells were sorted based on their c-kit and TLR2 expression, plated on a methylcellulose medium M3434 (StemCell Technologies) and assessed for their CFU potential at day 5 (EryP potential, left panel) and day 12 (multilineage potential, right panel). After 5 days of culture in M3434 supplemented with Epo and FCS, erythroid colonies derived from c-kit$^+$TLR2$^{+/-}$ were selected and their globin gene expression was assessed by qPCR (mean ± SEM; n=5-6; *$p \leq 0.05$; unpaired, two-tailed t-test). Representative primitive (EryP) and definitive (EryD) colonies are shown (left panel). After 12 days of culture in M3434, colonies derived from c-kit$^+$TLR2$^{+/-}$ cells were scored. Representative erythrocyte/megakaryocyte (E/Mk), granulocyte/monocyte (G/M) and mixed (Mix) colonies grown from c-kit$^+$TLR2$^+$ cells are shown.

**(c)** Gating strategy to determine the expression of Tie2, CD41 and CD31 on E7.5 *Actb*$^{EGFP+}$ Ter119$^-$ CD45$^-$ c-kit$^+$ and either TLR2$^+$ or TLR2$^-$ cells (two upper rows). CD31 median fluorescence is shown in the color-coded heatmap (middle row, two right plots). Tie2, CD41 and CD31 median fluorescence is shown in the t-SNE (t-distributed stochastic neighbour embedding) map (bottom row, three middle plots) displaying all *Actb*$^{EGFP+}$ Ter119$^-$ CD45$^-$ c-kit$^+$ cells (bottom row, green gate). The position of the Tie2$^+$ CD41$^+$ fraction of c-kit$^+$ TLR2$^-$ and c-kit$^+$ TLR2$^+$ subsets (blue and red gate from the middle and right plot in the middle row, respectively) in the t-SNE map is shown (bottom row, right plot).

**(d)** TLR2 triggered proliferation of E8.5 embryonic TLR2$^+$ c-kit$^+$ CD45$^-$ cells is MyD88-dependent. Proliferation of E8.5 embryonic *Actb*$^{EGFP+}$ TLR2$^+$ c-kit$^+$ CD45$^-$ cells derived from wt (MyD88$^{+/+}$) or MyD88 deficient (MyD88$^{-/-}$) mice in the absence (—) or presence (+) of Pam$_3$CSK$_4$ (1µg/ml, 72 hours) was assessed by FCM and quantified. Representative FCM plots are shown (mean ± SEM; n=3; *$p<0.05$, paired, two-tailed t-test).

Source data are provided as a Source Data file.

## Supplementary Figure 3

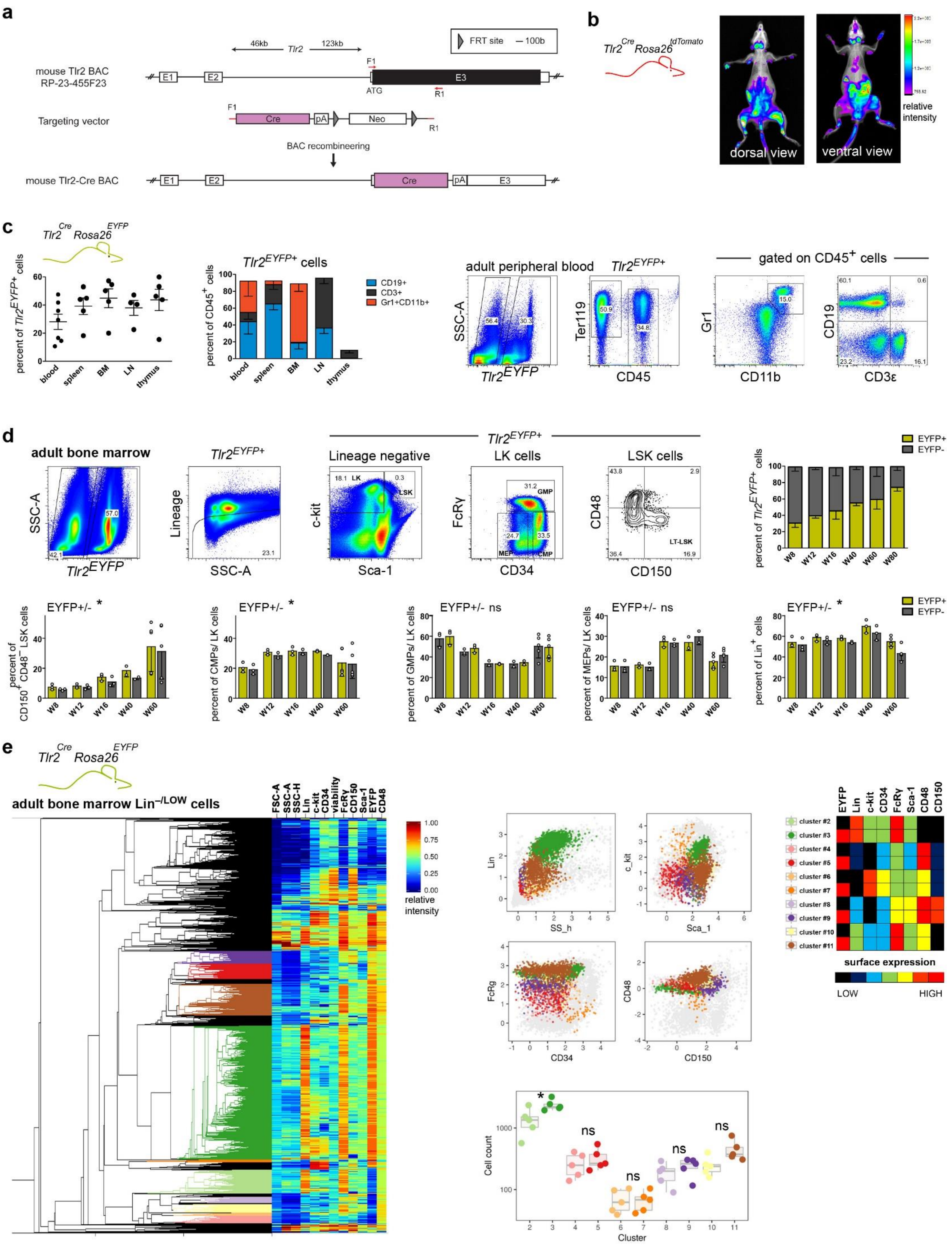

**Supplementary Figure 3**

**Lineage tracing shows strong labeling of adult hematopoietic cells in *Tlr2$^{Cre}$R26$^{EYFP}$* mice**

**(a)** Schematics of the generation of the *Tlr2$^{Cre}$* mouse strain by BAC recombineering.

**(b)** Representative images of the whole-body imaging of *Tlr2$^{Cre}$Rosa26$^{tdTomato}$* animals revealed *Tlr2* activity in the gut, brain, salivary glands, spleen, lymph nodes, and bones (presumably BM). tdTomato fluorescence was acquired using an Xtreme whole body imager (Bruker) on adult *Tlr2$^{Cre}$Rosa26$^{tdTomato}$* mice. The spectra of excitation wavelengths, 420nm to 540nm, with a 580-629nm emission filter were measured.

**(c)** FCM analysis of *Tlr2$^{Cre}$Rosa26$^{EYFP}$* adult tissues shows the contribution of *Tlr2$^{Cre}$*EYFP$^{+}$ cells to all hematopoietic lineages. The percentage of *Tlr2$^{Cre}$*EYFP$^{+}$ cells in hematopoietic organs and their contribution to the CD45$^{+}$ hematopoietic lineages in analyzed organs is shown (mean ± SEM; n=4-7). Representative FCM plots of adult peripheral blood are shown.

**(d)** FCM analysis of *Tlr2$^{Cre}$Rosa26$^{EYFP}$* adult bone marrow (BM) shows the contribution of *Tlr2$^{Cre}$*EYFP$^{+}$ cells to hematopoietic stem and progenitor populations, including LT-HSCs (Lin$^{-}$ Sca1$^{+}$ c-kit$^{+}$ CD150$^{+}$ CD48$^{-}$). The percentage of total *Tlr2$^{Cre}$*EYFP$^{+}$ cells in the BM increased with age. The percentage of different hematopoietic populations (CD150$^{+}$CD48$^{-}$LSKs, CMPs, GMPs, MEPs, Lin$^{+}$ cells) in EYFP$^{+}$ and EYFP$^{-}$ fractions of adult BM is shown (mean ± SD, n=3-5; *p<0.05; paired, two-tailed t-test, all EYFP$^{+}$ cells were tested against all EYFP$^{-}$ cells).

**(e)** Hierarchical clustering analysis (HCA) of FCM data acquired on adult BM Lin$^{-/LOW}$ cells from *Tlr2$^{Cre}$R26$^{EYFP}$* mice stained for indicated markers showed no bias in the labeling of hematopoietic progenitors, except for Lin$^{LOW}$ c-kit$^{INT}$ cells (green clusters). From the resulting hierarchy (left), the clusters (color coded cell populations) were selected based on dendrogram topology and a matrix of parametric scatterplots. The clusters formed log-normal distributed cell populations on the scatterplots (middle-top) and the cell abundance between EYFP positive and negative clusters (mean ± SEM; *p<0.05; Welch two sample t-test, middle-bottom) was then compared. The legend (right) describes the surface phenotype of selected clusters. Note, that clusters with the light and dark shade of the same color represents EYFP$^{-}$and EYFP$^{+}$ variant of cells with indicated phenotype, respectively. Data is representative of five experiments. Together, this data showed that *Tlr2*-driven EYFP labeling occurs at the basis of hematopoietic hierarchy and marks all lineages equally, suggesting that the activation of *Tlr2* locus also accompanies the emergence of LT-HSCs.

Source data are provided as a Source Data file.

**Supplementary Figure 4**

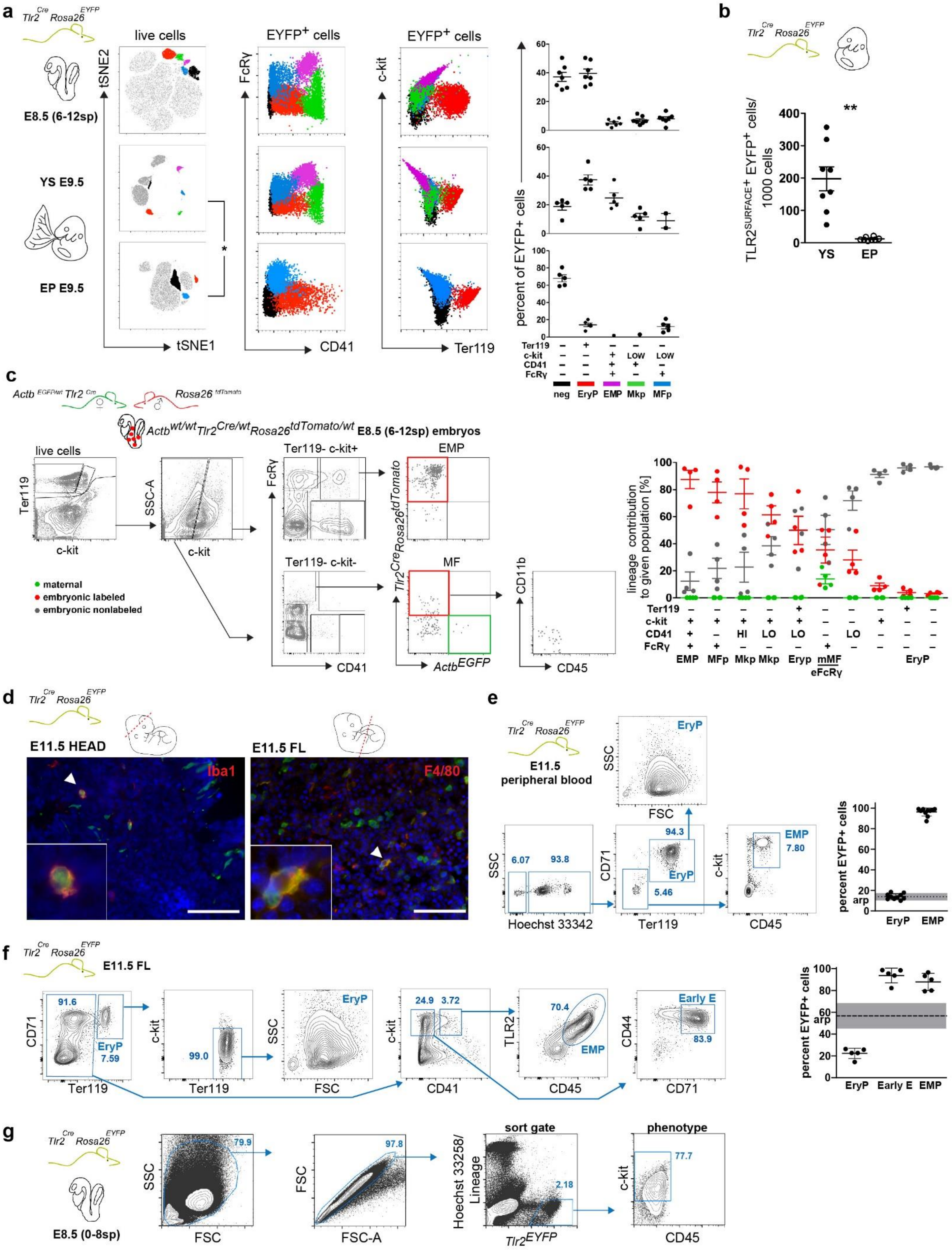

**Supplementary Figure 4**

**EYFP labeling of embryonic hematopoietic populations in *Tlr2$^{Cre}$Rosa26$^{EYFP}$* embryos**

**(a)** Five most abundant populations of *Tlr2$^{Cre}$*EYFP$^{+}$ cells in E8.5 and E9.5 *Tlr2$^{Cre}$Rosa26$^{EYFP}$* embryos (EP and YS) were identified by t-SNE analysis of FCM detection of surface antigens TLR2, c-kit, CD45, CD41, FcRγ and Ter119. The separate data for EP and YS was merged into one file for tSNE analysis (*) and the tissue of origin was implemented in the analysis. Scatter plots show the percentage of cells in each of *Tlr2$^{Cre}$*EYFP$^{+}$ clusters detected (EryP, primitive erythrocyte; EMP, erythro-myeloid progenitor; Mkp, Megakaryocyte progenitor; MFp, Macrophage progenitor; E8.75: n=7; E9.5: n=5; mean ± SEM).

**(b)** The relative number of TLR2$^{SURFACE+EYFP+}$ cells per 1000 cells in E9.5 YS and EP of *Tlr2$^{Cre}$ Rosa26$^{EYFP}$* embryos determined by FCM (YS and EP; mean ± SEM; n=8; **$p<0.01$; paired, two-tailed t-test).

**(c)** The frequencies of embryonic *Tlr2*-labeled, non-labeled cells and maternal cells were determined in E8.5 embryos by FCM of c-kit, CD41, FcRγ, and Ter119. Crosses between *Actb$^{EGFP/wt}$ Tlr2$^{Cre/Cre}$* females and *Rosa26$^{tdTomato/tdTomato}$* males facilitated the visualization of maternal *Actb*$^{EGFP+}$ cells (*Actb$^{EGFP/wt}$*, green) from embryonic *Actb$^{wt/wt}$ Tlr2$^{Cre/wt}$Rosa26$^{tdTomato/wt}$* *Tlr2*-non-labeled (grey) or *Tlr2*-labeled (red) cells in *Actb$^{wt/wt}$ Tlr2$^{Cre/wt}$Rosa26$^{tdTomato/wt}$* embryos (EMP, erythro-myeloid progenitor; Mk, megakaryocyte; mMF, maternal macrophage; eFcRγ, embryonic FcRγ cells; EryP, primitive erythrocyte; Mkp, Mk progenitor; MFp, MF progenitor; Eryp, erythroid progenitor; mean ± SEM; n=4). The gating hierarchy is shown.

**(d)** Cryosections of the head and FL from E11.5 *Tlr2$^{Cre}$Rosa26$^{EYFP}$* embryos stained for *Tlr2$^{Cre}$*EYFP (green) and co-stained with Iba1 or F4/80 (red), respectively. Nuclei were visualized by DAPI (blue). Sections were imaged with a Leica DM6000 microscope equipped with HCX PL APO 20x 0.7 and HCX PL APO 40x 0.75 objectives. Scale bars represent 50μm. Representative images are shown (n=3 independent experiments with 1-2 embryos of each genotype per experiment).

**(e)** FCM analysis of peripheral blood (PB) collected from E11.5 *Tlr2$^{Cre}$Rosa26$^{EYFP}$* embryos. While circulating EMPs are efficiently labeled with EYFP, primitive erythroid cells (EryP) are labeled on background level (arp).

**(f)** FCM analysis of FLs isolated from the E11.5 *Tlr2$^{Cre}$Rosa26$^{EYFP}$* embryos as in **(e)**. Activation of *Tlr2* locus efficiently labels EMPs and early erythroid progenitors (Early E) in E11.5 FL, while EryPs are inefficiently labeled below arp.

**(g)** Gating strategy for sorting of E8.5 *Tlr2$^{Cre}$*EYFP$^{+}$ Lin$^{-}$ embryonic cellsusing c-kit and CD45 markers.

Source data are provided as a Source Data file.

## Supplementary Figure 5

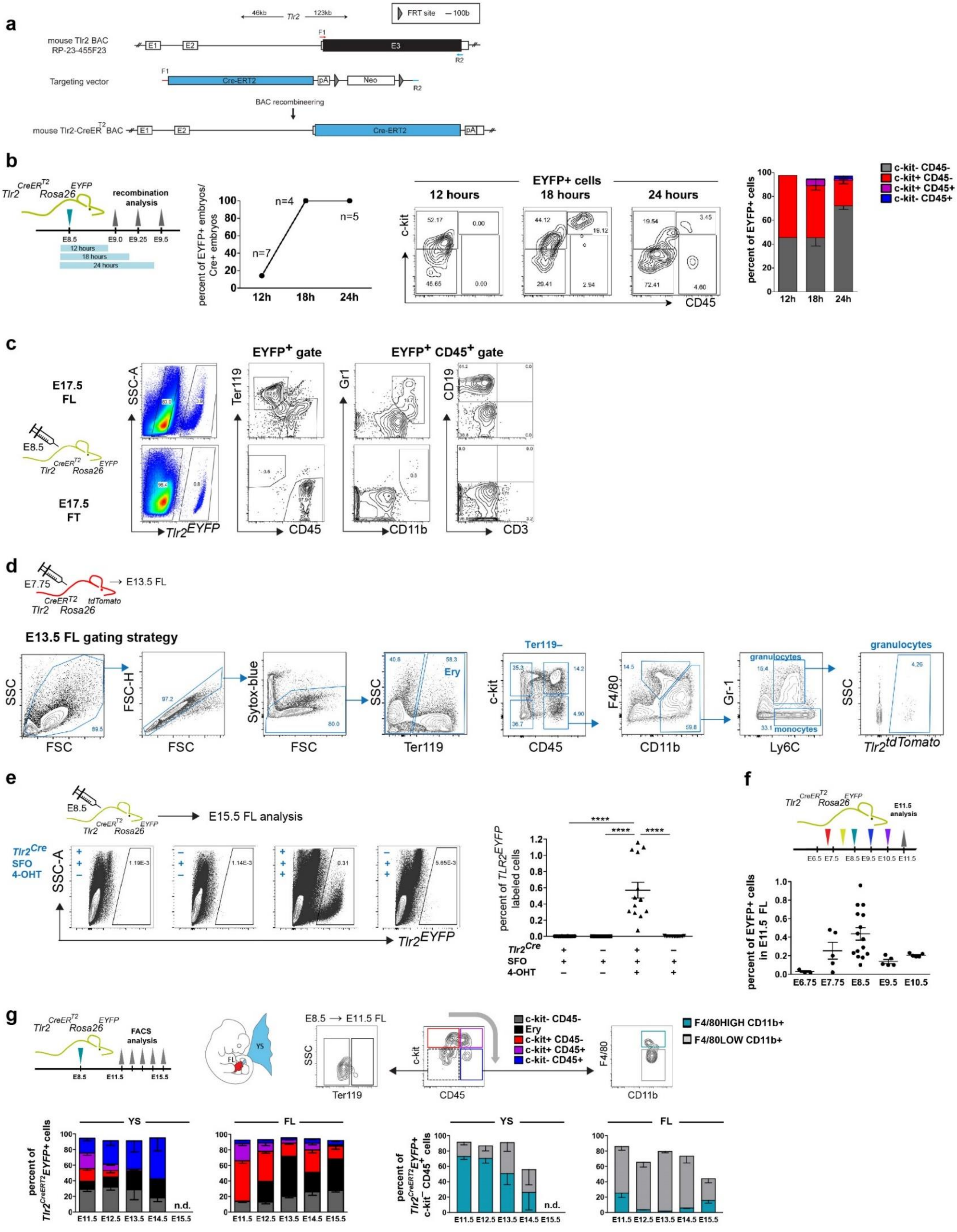

**Supplementary Figure 5**

**EYFP labeling of embryonic hematopoietic populations in *Tlr2$^{CreERT2}$Rosa26$^{EYFP}$* embryos**

**(a)** Generation of *Tlr2$^{CreERT2}$* mouse strain by BAC recombineering.

**(b)** Labeling efficiency was assessed 12-24 hours after administration of 4-OHT to E8.5 *Tlr2$^{CreERT2}$Rosa26$^{EYFP}$* embryos. Phenotypic analysis of recombined cells is shown (mean ± SEM; n=4-7).

**(c)** The hematopoietic fate of *Tlr2$^{CreERT2}$*EYFP$^{+}$ cells pulsed with one dose of 4-OHT at E8.5 was determined in E17.5 FL and FT; representative FCM analysis with the gating strategy.

**(d)** Gating hierarchy to determine the labeling efficiency of different hematopoietic populations including granulocytes and monocytes in E13.5 *Tlr2$^{CreERT2}$Rosa26$^{tdTomato}$* FL upon administration of a single dose of 4-OHT at E7.75.

**(e)** To rule out potential leakage of the *CreERT2* system, 4-hydroxytamoxifen (4-OHT, 1.5 mg) or the vehicle alone, (sunflower oil with ethanol (SFO)) was administered to pregnant mice at E8.5 i.p. and the frequencies of EYFP labeled cells were determined in E15.5 FL (mean ± SEM; n=13-17; **** p≤0.0001; unpaired, two tailed t-test).

**(f)** *Tlr2$^{CreERT2}$Rosa26$^{EYFP}$* embryos were pulsed from E6.75 to E10.5 by a single dose of 4-OHT and the percentage of *Tlr2$^{CreERT2}$*EYFP$^{+}$ cells was determined in E11.5 FL (mean ± SEM; n=4-15).

**(g)** Cell fate of *Tlr2$^{CreERT2}$*EYFP$^{+}$ cells labeled at E8.5 by a single dose of 4-OHT and analyzed by FCM at E11.5-E15.5 (mean ± SEM; n=4-15); n.d.=not detectable.

Source data are provided as a Source Data file.

**Supplementary Figure 6**

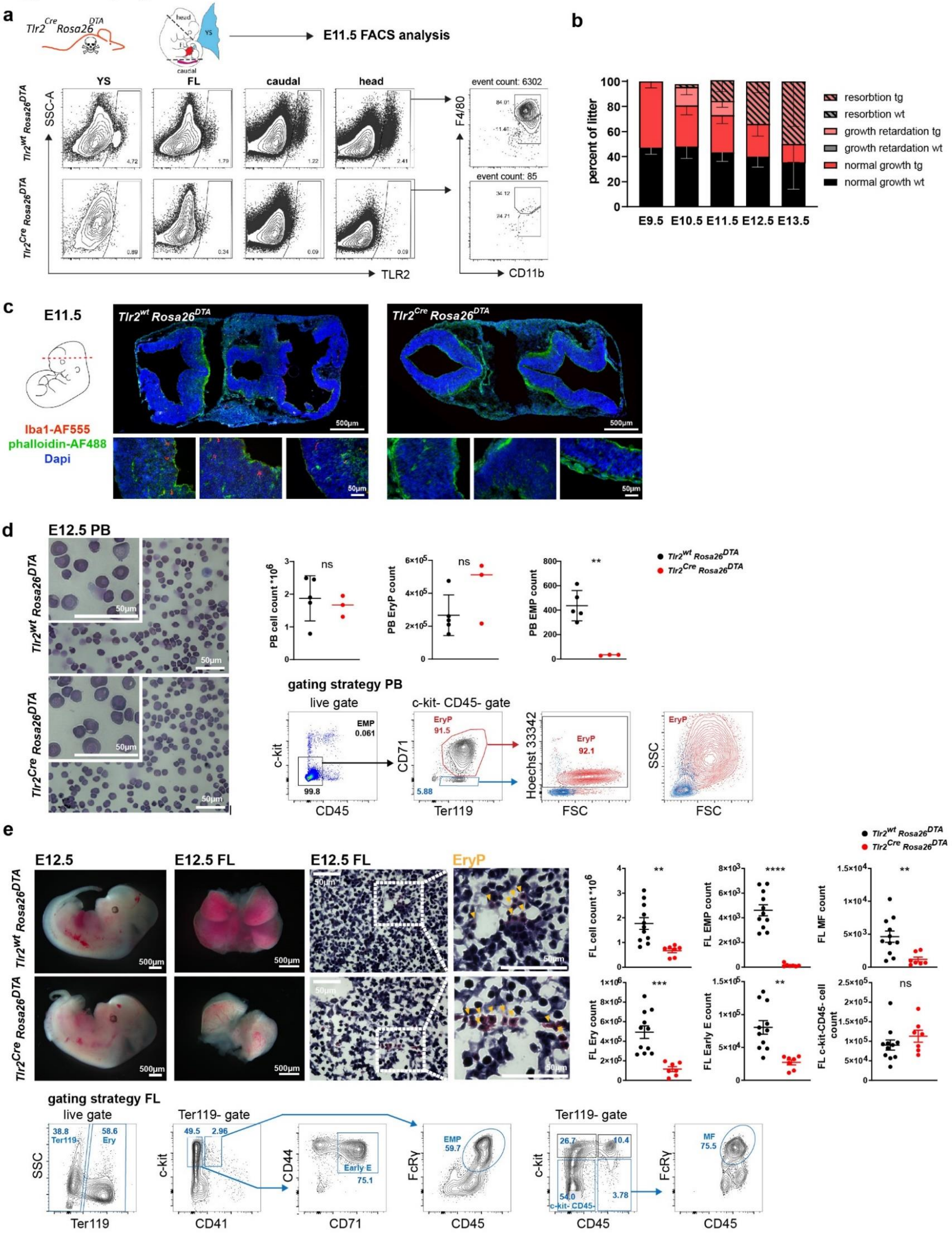

**Supplementary Figure 6**

**Primitive hematopoiesis remains intact upon deletion of TLR2⁺ cells in *Tlr2^Cre^Rosa26^DTA^* embryos**

**(a)** The presence of $TLR2^{+}$ cells in the indicated tissues of E11.5 $Tlr2^{wt}Rosa26^{DTA}$ (upper panel) and $Tlr2^{Cre}Rosa26^{DTA}$ (lower panel) embryos was determined by FCM. Representative data from three independent experiments are shown.

**(b)** Depletion of $TLR2^{+}$ cells from $Tlr2^{Cre}Rosa26^{DTA}$ embryos leads to embryonic lethality before E13.5. $Rosa26^{DTA/DTA}$ females were crossed with $Tlr2^{Cre/wt}$ males to obtain litters with equal numbers of wt ($Tlr2^{wt/wt}Rosa26^{DTA/wt}$) and tg ($Tlr2^{Cre/wt}Rosa26^{DTA/wt}$) genotypes if no lethality occurs. Graph shows the proportion of normally growing embryos, embryos with growth retardation and resorbed embryos at indicated days p.c. in both wt and transgenic embryos of individual litters (mean ± SEM; n=2-10).

**(c)** Transverse cryosections of E11.5 $Tlr2^{wt}Rosa26^{DTA}$ (left panel) and $Tlr2^{Cre}Rosa26^{DTA}$ (right panel) embryos taken from the brain were stained for the microglial marker, Iba1 (red) and actin (green); nuclei were visualized with DAPI (blue). A representative image is shown (n=3 independent experiments with 1-2 embryos of each genotype per experiment).

**(d)** Analysis of peripheral blood harvested from E12.5 $Tlr2^{wt}Rosa26^{DTA}$ and $Tlr2^{Cre}Rosa26^{DTA}$ embryos. MGG staining shows abundant EryPs in both, $Tlr2^{wt}Rosa26^{DTA}$ and $Tlr2^{Cre}Rosa26^{DTA}$ embryos (left panel). A representative image is shown (n=5 independent experiments with 1-4 embryos of each genotype per experiment). FCM analysis shows no differences in PB cellularity and EryP counts in $Tlr2^{wt}Rosa26^{DTA}$ and $Tlr2^{Cre}Rosa26^{DTA}$ embryos, whereas circulating EMPs are specifically deleted in $Tlr2^{Cre}Rosa26^{DTA}$ PB (mean ± SD; n=3-5; ** $p \leq 0.01$; unpaired, two tailed t-test).

**(e)** At E12.5 some $Tlr2^{Cre}Rosa26^{DTA}$ embryos still display no morphological differences from $Tlr2^{wt}Rosa26^{DTA}$ embryos. However, their FLs are pale and show reduced cellularity. A representative image is shown (n=5 independent experiments with 1-4 embryos of each genotype per experiment). EMPs and MFs are depleted and the numbers of erythrocytes (Ery) as well as the early erythroid progenitors (Early E) are reduced in $Tlr2^{Cre}Rosa26^{DTA}$ FLs (mean ± SEM; n=7-11; ** $p \leq 0.01$, *** $p \leq 0.001$, **** $p \leq 0.0001$; unpaired, two tailed t-test). Despite these observations, EryPs (yellow arrows) are present in FL of both $Tlr2^{wt}Rosa26^{DTA}$ and $Tlr2^{Cre}Rosa26^{DTA}$ embryos (middle panel).

Source data are provided as a Source Data file.

## Supplementary Figure 7

**a**

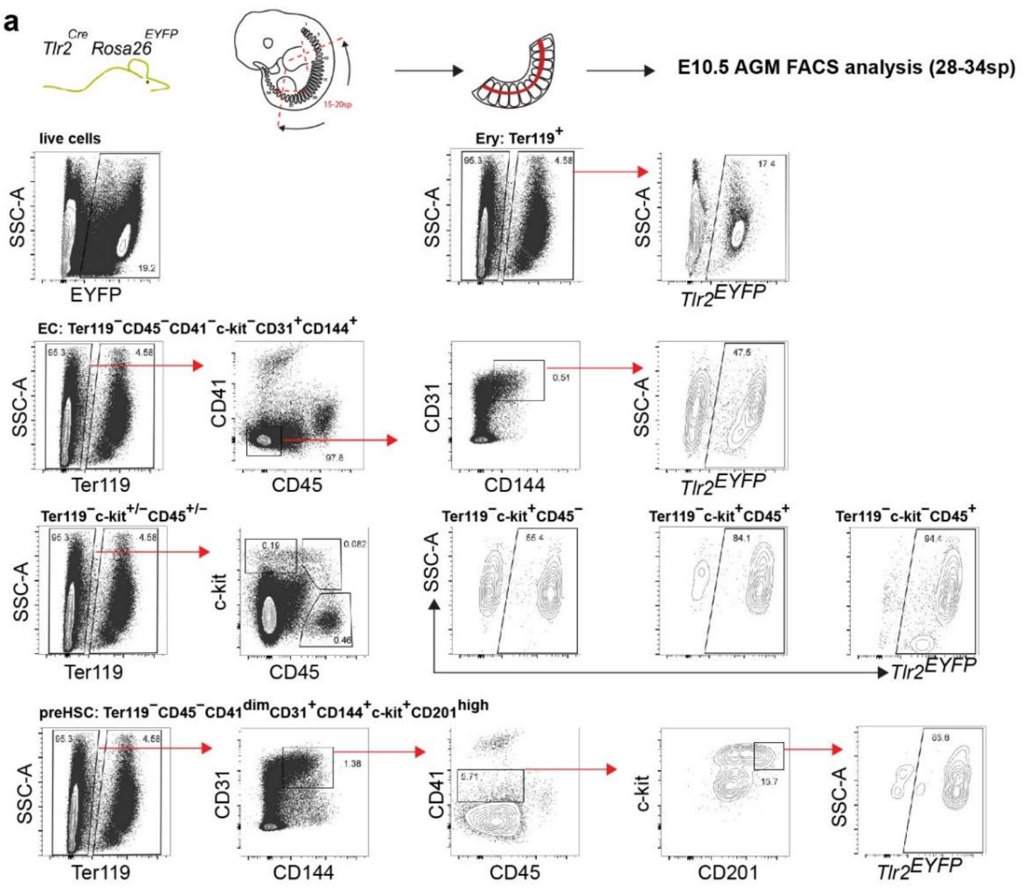


**b**

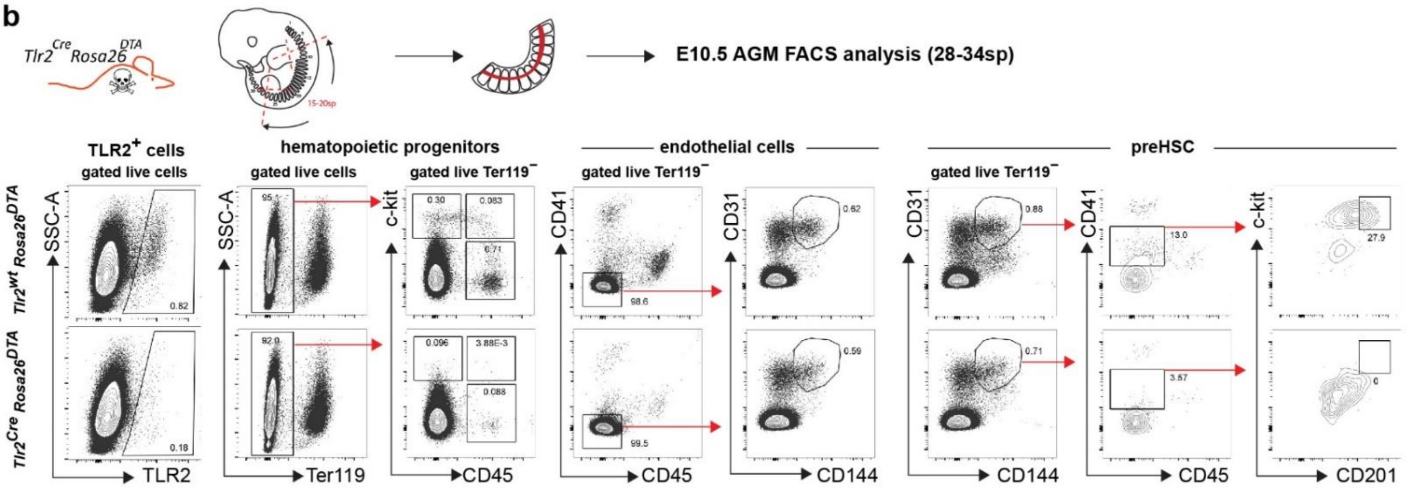

**Supplementary Figure 7**

**Gating strategies to identify pre-HSCs in AGM of *Tlr2$^{Cre}$Rosa26$^{EYFP}$* and *Tlr2$^{Cre}$Rosa26$^{DTA}$* embryos**

**(a)** Aortic regions were dissected from E10.5 *Tlr2$^{Cre}$Rosa26$^{EYFP}$* embryos (28-34sp) and different hematopoietic populations as well as endothelial cells were analyzed for their recombination efficiency by FCM. The gating strategies for individual cell populations are indicated.

**(b)** Aortic regions were dissected from E10.5 *Tlr2$^{Cre}$Rosa26$^{DTA}$* embryos (28-34sp) and the frequencies of hematopoietic and endothelial cells were assessed by FCM. The gating strategies for individual cell populations are indicated.

## Supplementary Figure 8

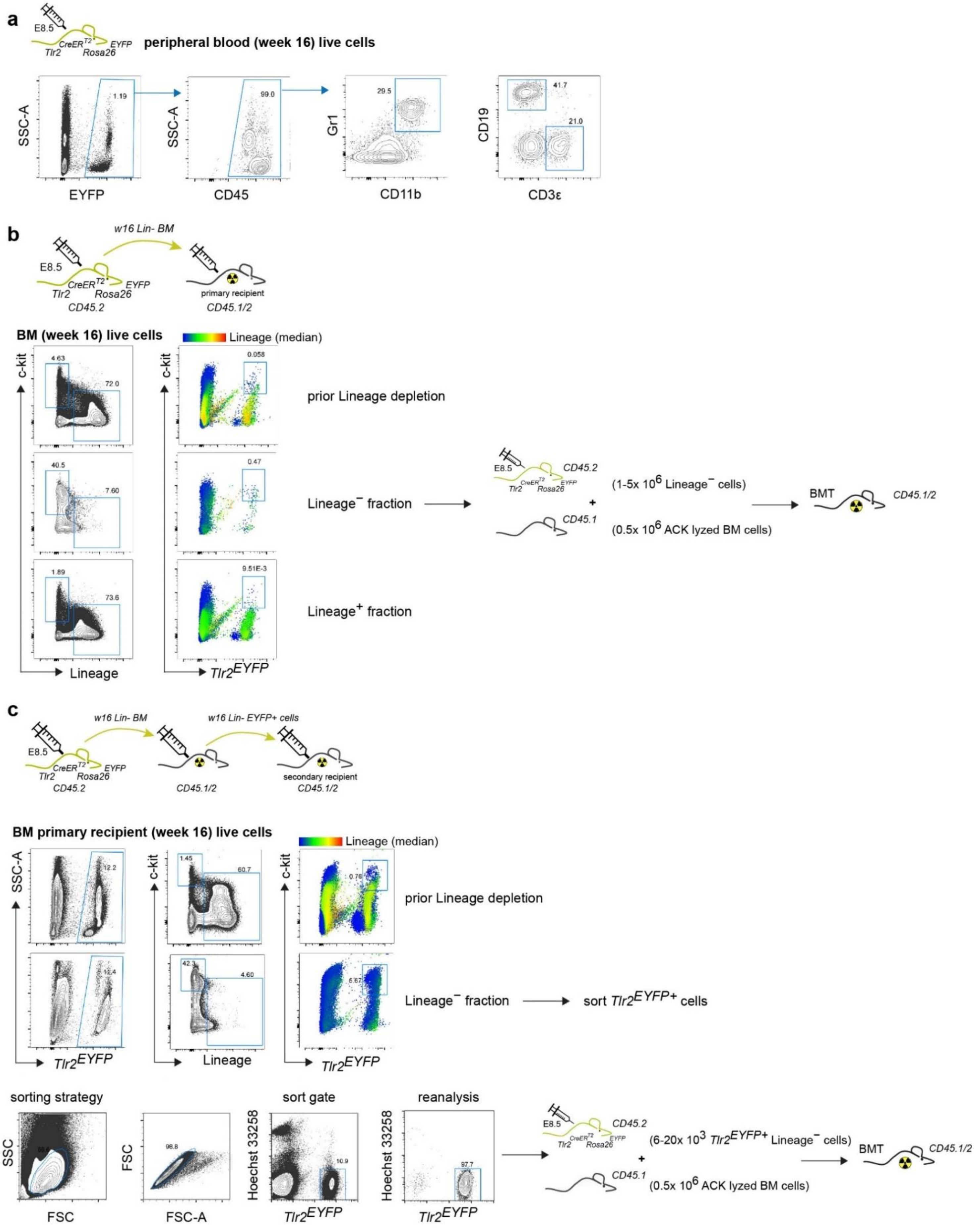

**Supplementary Figure 8**

**Contribution of E8.5 *Tlr2* labeled hematopoietic progenitors to adult hematopoiesis**

**(a)** FCM analysis of hematopoietic lineages in peripheral blood of adult animals labeled at E8.5.

**(b)** FCM analysis of lineage depleted BM cells isolated from adult animals labeled at E8.5.

**(c)** BM cells isolated from adult primary recipients that received $Lin^{-}$ BM from adult animals labeled at E8.5 were lineage depleted using magnetic beads. Before their transfer to secondary recipients, $EYFP^{+}$ cells were sorted as indicated.

**Supplementary Table 1.**

**List of primer sequences used for RT-qPCR analysis of selected genes**

| Gene | Forward primer 5'– 3' | Reverse primer 5'– 3' |
|---|---|---|
| *CasC3* | TTCGAGGTGTGCCTAACCA | GCTTAGCTCGACCACTCTGG |
| *Cd11b* | CAATAGCCAGCCTCAGTGC | GAGCCCAGGGGAGAAGTG |
| *Cd31* | GCTGGTGCTCTATGCAAGC | ATGGATGCTGTTGATGGTGA |
| *Cd34* | GGGTAGCTCTCTGCCTGATG | TCCGTGGTAGCAGAAGTCAA |
| *Cd41* | TGCTGCTGACCCTGCTAGT | GTCGATTCCGCTTGAAGAAG |
| *Cd45* | AGTAGGAAACTTGCTCCCCATCTGA | TGGAGATCAGCTGTGCCCCT |
| *Cd93* | GAGAATCAGTACAGCCCAACACCAG | TGCCTATCCCAAGCCACCAGGT |
| *Cd105* | CGTTCTCTTCCTGGATTTTCC | ATTTTGCTTGGATGCCTGAA |
| *Cd144* | GTTCAAGTTTGCCCTGAAGAA | GTGATGTTGGCGGTGTTGT |
| *Cd150* | GACCCCTGCACAACCATT | TGTGGTGGGGTTTGGTTC |
| *Cd244* | TTGTACCGTGTACGAGGAGGT | CAAAGTTCTCCAGCTCTCTGC |
| *c-kit* | GGAGCCCACAATAGATTGGTAT | CACTGGTGAGACAGGAGTGG |
| *Csf1r* | CGAGGGAGACTCCAGCTACA | GACTGGAGAAGCCACTGTCC |
| *Flk1* | CCCCAAATTCCATTATGACAA | CGGCTCTTTCGCTTACTGTT |
| *Gapdh* | CGTCCCGTAGACAAAATGGT | TTGATGGCAACAATCTCCAC |
| *Gata1* | TCCCAGTCCTTTCTTCTCTCC | TCACACACTCTCTGGCCTCA |
| *Gata2* | GCTTCACCCCTAAGCAGAGA | TGGCACCACAGTTGACACA |
| *Hbb-bh1* | TGGATCCTGAGAACTTCAAGC | ATTGGCCACTCCAATCACC |
| *Hbb-b1* | TGCATGTGGATCCTGAGAAC | GTGAAATCCTTGCCCAGGT |
| *Lmo2* | CGAAAGGAAGAGCCTGGAC | CGGTCCCCTATGTTCTGCT |
| *Mal* | CTGCACTATGGCTTCATCCTC | AGAGCCTGCCTGAACCAGT |
| *Mpo* | GGAAGGAGACCTAGAGGTTGG | TAGCACAGGAAGGCCAATG |
| *MyD88* | TGGCCTTGTTAGACCGTGA | AAGTATTTCTGGCAGTCCTCCTC |
| *Runx1* | CTCCGTGCTACCCACTCACT | GCACTCTGGTCACCGTCAT |
| *Sarm1* | GCTTGCTGGAGCAGATCCT | TCACGCCTAGACCGATGC |
| *Tie2* | GGCCAGGTACATAGGAGGAA | CCCACTTCTGAGCTTCACATC |
| *Tlr1* | GGCGAGCAGAGGCAATTGTGGA | TTCTTCAGAGCATTGCCACATGGG |
| *Tlr2* | GTTCATCTCTGGAGCATCCG | ACTCCTGAGCAGAACAGCGT |
| *Tlr3* | ATACAGGGATTGCACCCATA | CCCCCAAAGGAGTACATTAGA |
| *Tlr4* | GGACTCTGATCATGGCACTG | CTGATCCATGCATTGGTAGGT |
| *Tlr5* | ACGCACCACACTTCAGCA | AGCCTCGGAAAAAGGCTATC |
| *Tlr6* | GTCAGTGCTGGAAATAGAGC | CCACGATGGGTTTTCTGTCT |
| *Tlr7* | ACTCTCTTCTCCTCCACCAGACCT | GAGGCCACTTGTCTGTCACACTG |
| *Tlr8* | CAAACGTTTTACCTTCCTTTGTC | ATGGAAGATGGCACTGGTTC |
| *Tlr9* | CGCCCAGTTTGTCAGAGGGAGC | GGTGCAGAGTCCTTCGACGGAG |
| *Tlr11* | ATGGGGCTTTATCCCTTTTG | AGATGTTATTGCCACTCAACCA |
| *Tlr12* | TTTCAAGCACTGGCCTAACC | GAAGCCTAGGCATGGCAGT |
| *Tlr13* | CTATGTGCTAGGAGCTTCTGAGAG | AGGAAGCAGAGAACCAGGAA |
| *Tram* | TGGTCAAGCAGTACCACTTCC | GAGACGCCTTAGCCTCCAGT |
| *Trif* | ACCAGGGACCGGGAGATCTACCA | CAAAGATGCTGGAGGGCGGCA |
