# Supplementary material for: Toll-like receptor 2 expression on c-kit+ cells tracks the emergence of embryonic definitive hematopoietic progenitors
Source: Nat Commun. 2019 Nov 15;10:5176. doi: 10.1038/s41467-019-13150-0 (PMC6858454; doi:10.1038/s41467-019-13150-0)
Supplement: Supplementary file 3 — Reporting Summary [file 41467_2019_13150_MOESM3_ESM.pdf]

nature research

Corresponding author(s): Dominik Filipp

Last updated by author(s): Aug 26, 2019

Reporting Summary

Nature Research wishes to improve the reproducibility of the work that we publish. This form provides structure for consistency and transparency in reporting. For further information on Nature Research policies, see Authors & Referees and the Editorial Policy Checklist .

Statistics

For all statistical analyses, confirm that the following items are present in the figure legend, table legend, main text, or Methods section.

n/a	Confirmed	
☐	☒	The exact sample size (*n*) for each experimental group/condition, given as a discrete number and unit of measurement
☐	☒	A statement on whether measurements were taken from distinct samples or whether the same sample was measured repeatedly
☐	☒	The statistical test(s) used AND whether they are one- or two-sided<br>*Only common tests should be described solely by name; describe more complex techniques in the Methods section.*
☒	☐	A description of all covariates tested
☐	☒	A description of any assumptions or corrections, such as tests of normality and adjustment for multiple comparisons
☐	☒	A full description of the statistical parameters including central tendency (e.g. means) or other basic estimates (e.g. regression coefficient) AND variation (e.g. standard deviation) or associated estimates of uncertainty (e.g. confidence intervals)
☐	☒	For null hypothesis testing, the test statistic (e.g. *F*, *t*, *r*) with confidence intervals, effect sizes, degrees of freedom and *P* value noted<br>*Give P values as exact values whenever suitable.*
☒	☐	For Bayesian analysis, information on the choice of priors and Markov chain Monte Carlo settings
☒	☐	For hierarchical and complex designs, identification of the appropriate level for tests and full reporting of outcomes
☒	☐	Estimates of effect sizes (e.g. Cohen's *d*, Pearson's *r*), indicating how they were calculated

Our web collection on statistics for biologists contains articles on many of the points above.

Software and code

Policy information about availability of computer code

Data collection	BD FACSDIVA™ SOFTWARE, Roche LightCycler 480 1.5 software, NIS-Elements, Imaris 7.3
Data analysis	GraphPad Prism, FlowJo V10, LAS AF Lite, NIS-Elements, Imaris 7.3, Roche LightCycler 480 1.5 software, R version 3.3.3 (2017-03-06) https://cran.r-project.org/, hca algotrithm (10.1002/cyto.a.21148), Barnes-Hut implementation of t-Distributed Stochastic Neighbour Embedding (t-SNE) https://github.com/jkrijthe/Rtsne

For manuscripts utilizing custom algorithms or software that are central to the research but not yet described in published literature, software must be made available to editors/reviewers. We strongly encourage code deposition in a community repository (e.g. GitHub). See the Nature Research guidelines for submitting code & software for further information.

Data

Policy information about availability of data

All manuscripts must include a data availability statement. This statement should provide the following information, where applicable:
- Accession codes, unique identifiers, or web links for publicly available datasets
- A list of figures that have associated raw data
- A description of any restrictions on data availability

Data are available from corresponding author upon request.

Field-specific reporting

Please select the one below that is the best fit for your research. If you are not sure, read the appropriate sections before making your selection.

Life sciences study design

All studies must disclose on these points even when the disclosure is negative.

Sample size	No statistical methods were used to predetermine sample size. Sample size were not calculated prior to performing experiment. To minimized the number of mice models used, sample size were determined to be sufficient to detect differences between groups. All comparisons had minimum of 3 data points. Non of the data points used was generated as technical replicate.
Data exclusions	No data were excluded from the study.
Replication	All attempts for replication were successful.
Randomization	Animals were randomly selected for experiments.
Blinding	The investigators were not blinded to allocation during following experiments and outcome assessment.

Reporting for specific materials, systems and methods

We require information from authors about some types of materials, experimental systems and methods used in many studies. Here, indicate whether each material, system or method listed is relevant to your study. If you are not sure if a list item applies to your research, read the appropriate section before selecting a response.

Materials & experimental systems

n/a	Involved in the study	
☐	☒	Antibodies
☐	☒	Eukaryotic cell lines
☒	☐	Palaeontology
☐	☒	Animals and other organisms
☒	☐	Human research participants
☒	☐	Clinical data

Methods

n/a	Involved in the study	
☒	☐	ChIP-seq
☐	☒	Flow cytometry
☒	☐	MRI-based neuroimaging

Antibodies

Antibodies used

Anti-mouse B220-PB Biolegend RA3-6B2
Anti-mouse B220-Alexa647 Biolegend RA3-6B2
Anti-mouse CD3ε-biotin Biolegend 17A2
Anti-mouse CD3ε-PB Biolegend 17A2
Anti-mouse CD3ε-PerCPCy5.5 Biolegend 17A2
Anti-mouse CD11b-biotin eBioscience M1/70
Anti-mouse CD11b-PE eBioscience M1/70
Anti-mouse CD11b-Alexa700 Sony M1/70
Anti-mouse CD19-biotin Biolegend 6D5
Anti-mouse CD19-APC Biolegend 6D5
Anti-mouse CD31-APC Biolegend 390
Anti-mouse CD31-FITC Biolegend MEC13.3
Anti-mouse CD31-PerCP-eFluor710 eBioscience 390
Anti-mouse CD31-purified, rat polyclonal BD Pharmingen Cat#550274
Anti-mouse CD34-Alexa700 eBioscience RAM34
Anti-mouse CD41-APC-Cy7 eBioscience MWReg30
Anti-mouse CD45-Alexa700 eBioscience 30-F11
Anti-mouse CD45- PerCPCy5.5 Biolegend 30-F11
Anti-mouse CD45-Pacific Blue Biolegend 30-F11
Anti-mouse CD45.1-PECy7 Biolegend A20
Anti-mouse CD45.2-PE eBioscience 104
Anti-mouse CD48-biotin eBioscience HM48-1
Anti-mouse CD144-PECy7 Biolegend BV13
Anti-mouse CD150-PECy7 Biolegend TC15-12F12.2
Anti-mouse CD201- PerCP-eFluor710 eBioscience eBio1560
Anti-mouse c-kit-PE Biolegend ACK2
Anti-mouse c-kit-APC eBioscience ACK2
Anti-mouse c-kit-Alexa700 eBioscience ACK2
Anti-mouse c-kit, goat RaD Systems Cat#AF1356

Anti-mouse F4/80, purified Biorad Cl:A3-1 Cat#MCA497GA
Anti-mouse F4/80-PECy7 eBioscience BM8
Anti-mouse FcRγ, purified, rat Biolegend 2.4G2
Anti-mouse FcRγ-PE Biolegend 93
Anti-mouse FcRγ-PECy7 Biolegend 93
Anti-GFP, purified rabbit polyclonal Thermo Fisher Scientific Cat#A11122
Anti-mouse Gr-1-biotin Biolegend RB6-8C5
Anti-mouse Gr-1-PB Biolegend RB6-8C5
Anti-mouse CD44-PE Biolegend MI7 Cat#103008
Anti-mouse CD71-APC Biolegend RI7217 Cat#113820
Anti-mouse CD41-FITC Biolegend MWReg30 Cat#133904
Anti-mouse Gr-1-PE Biolegend RB6-8C5
Anti-mouse Gr-1-Alexa647 Biolegend RB6-8C5
Anti-mouse Iba1, purified, goat polyclonal Abcam Cat#ab5076
Isotype control-Alexa647, mouse Sony MOPC-21
Anti-mouse Lineage-biotin Biolegend Cat# 133307
Anti-mouse Lineage-Pacific Blue Biolegend Cat# 133306
Anti-mouse Ly6C-FITC BD Pharmingen AL-21
Anti-mouse Ly6G-BV421 BD Pharmingen 1A8
Anti-mouse Sca1-PerCP Biolegend D7
Anti-mouse Tie2-PE eBioscience TEK4
Anti-mouse Ter119-PB Biolegend TER-119
Anti-mouse Ter119-PECy7 Biolegend TER-119
Anti-mouse Ter119-Briliant Violet 605 Biolegend TER-119
Anti-mouse TLR2-purified eBioscience 6C2
Anti-mouse TLR2-biotin Biolegend T2.5
Anti-mouse TLR2-Alexa647 Biolegend T2.5
Streptavidin-APC Biolegend Cat#405207
Streptavidin-APC-Cy7 Biolegend Cat#405208
Streptavidin-PECy7 Biolegend Cat#405206
Streptavidin-Qdot605 eBioscience Cat#93-4317-41
Chicken anti goat-A488 Life Technologies Cat#A-21467
Chicken anti rat-A647 Life Technologies Cat#A-21472
Donkey anti rat-Alexa594 Life Technologies Cat#A-21209
Donkey anti rabbit-Alexa488 Life Technologies Cat#A21206
Donkey anti goat-Alexa555 Life Technologies Cat#A21432
Donkey anti goat-Alexa568 Life Technologies Cat#A-11057
Goat anti rat-Alexa488 Life Technologies Cat#A11006
Goat anti rat-Alexa568 Life Technologies Cat#A11077
Anti-biotin MicroBeads Ultrapure Miltenyi Biotech Cat#130-105-637

Validation

All antibodies were titrated and used based on manufacturer recommendations.

Eukaryotic cell lines

Policy information about cell lines

Cell line source(s)

OP-9 stromal cells (Nakano et al. 1994. Science 265: 1098-1101) were a gift from J.C. Zuniga-Pflucker, ST-2 stromal cells (Ogawa et al. 1988. Embo j. 7: 1337-134) were a gift from prof. Ludger Klein.

Authentication

None of the cell lines used were authenticated.

Mycoplasma contamination

All cell lines were tested for Mycoplasma by PCR on a regular basis. All cell lines were negative for Mycoplasma contamination.

Commonly misidentified lines
(See ICLAC register)

No commonly misidentified cell lines were used in this study.

Animals and other organisms

Policy information about studies involving animals; ARRIVE guidelines recommended for reporting animal research

Laboratory animals

All experiments were done on mice (Mus musculus domesticus). For experiments where time pregnant females were used to obtain embryos at specific gestational stage, females (CD1, C57Bl/6J, ActbEGFP, Rosa26EYFP, Rosa26tdTomato, RosaDTA, 8-16week of age) were mated with proven male breeders (C57Bl/6J, ActbEGFP, Tlr2Cre, Tlr2CreERT2, Tlr2CreActbEGFP, RosatdTomato, 8-40 weeks of age). To determine recombination efficiency, adult 8-60-week old Rosa26EYFPTlr2Cre animals

	were used. For BMT experiments, 8-12 week old C57Bl/6JxLy5.1 females were lethally irradiated and injected with BM derived from 16-week old RosaEYFPTlr2CreERT2 animals, where recombination was induced by a single 4-OHT pulse at a specific embryonic stage. 500,000 BM cells from Ly5.1 animals were co-injected. 16 weeks after BMT, mice were sacrificed and BM was transferred to 8-12week old lethally irradiated secondary recipients together with BM from 8-10week old Ly5.1 animals. Description of research mice used for experiments can be found in the Methods.
Wild animals	No wild animals were used in this study.
Field-collected samples	The study did not involve samples collected from the field.
Ethics oversight	All experiments were approved by the Ministry of Agriculture of the Czech Republic and the ethical committee of the Institute of Molecular Genetics.

Note that full information on the approval of the study protocol must also be provided in the manuscript.

Flow Cytometry

Plots

Confirm that:

☐ The axis labels state the marker and fluorochrome used (e.g. CD4-FITC).

☐ The axis scales are clearly visible. Include numbers along axes only for bottom left plot of group (a 'group' is an analysis of identical markers).

☒ All plots are contour plots with outliers or pseudocolor plots.

☒ A numerical value for number of cells or percentage (with statistics) is provided.

Methodology

Sample preparation	For flow cytometry experiments and cell sorting, organs, embryos or yolk sacs were incubated in 1mg/ml dispase (Invitrogen) in HBSS for 10 minutes at 37°C with occasional gentle pipetting. Digestion was stopped by the washing in 2% FCS in HBSS. Cell suspensions were then passed through a 50µm cell strainer. Cell suspensions were centrifuged at 370g for 7 min. If necessary red blood cells were lysed in ACK solution. Cell suspensions were resuspended in FACS buffer (PBS, 3 % FCS, 2 mM EDTA). After Fc receptor blocking (in experiments where FcRγ was not stained) single cell suspensions were stained with conjugated monoclonal antibodies for 30 minutes on ice.<br>Peripheral blood of adult animals was collected from the facial vein to PBS EDTA solution and red blood cells were lysed in ACK solution.<br>Sample preparation is described in the Method section .
Instrument	LSRII flow cytometer (BD Biosciences), LSR Fortessa SORP (647800E6, BD Biosciences), Influx cell sorter (BD Biosciences)
Software	BD FACSDIVA™ SOFTWARE,), Version 8.0.1; dailly calibrations were performed using the built-in CS&T software module with CS&T beads (Becton, Dickinson and Company, BD Biosciences, USA). Bead lot ID: 72518 (RUO).
Cell population abundance	When possible, the post-sorting analysis was performed, with an average cell purity above 98%. For qRT-PCR analyses, cells were sorted directly to RNA lysis buffer. For clonogenic cell assays, all cells were sorted directly to M3434 and plated upon vortexing. Sample preparation is described in the Method section.
Gating strategy	Gating strategies for individual experiments are depicted in respective Figures or Supplementary Figures. Boundaries between positive and negative populations were determined based on unstained and FMO and isotype controls.

☒ Tick this box to confirm that a figure exemplifying the gating strategy is provided in the Supplementary Information.
