# Supplementary material for: Toll-like receptor 2 expression on c-kit+ cells tracks the emergence of embryonic definitive hematopoietic progenitors
Source: Nat Commun. 2019 Nov 15;10:5176. doi: 10.1038/s41467-019-13150-0 (PMC6858454; doi:10.1038/s41467-019-13150-0)
Supplement: Supplementary file 4 — Description of Additional Supplementary Files [file 41467_2019_13150_MOESM4_ESM.pdf]

**Title:** Supplemental Movie 1

**Description:** (Related to Figure 1). Anatomical location of TLR2+ cells in E7.5 embryo. 3D reconstructed image of E7.5 embryo stained with anti-TLR2 antibody and counterstained with DAPI. Signals were visualized and digital images were obtained using a Zeiss LSM 780 equipped with two photon, argon and helium–neon lasers.
